# Using IBD-REFER: A Substitute for Clinical Judgement?

**DOI:** 10.1093/crocol/otaa028

**Published:** 2020-04-24

**Authors:** Jeffrey M Dueker, Joel R Rosh

**Affiliations:** 1 Assistant Professor of Medicine, Division of Gastroenterology, Hepatology and Nutrition, University of Pittsburgh Medical Center; 2 Pediatric Gastroenterology and Vice Chairman, Clinical Development and Research Affairs, Goryeb Children’s Hospital/Atlantic Health, Professor of Pediatrics, Icahn School of Medicine at Mount Sinai

The need for early, accurate identification of patients with inflammatory bowel disease (IBD) has never been more important, especially with rising incidence of IBD^1^ and the association of improved outcomes for patients treated early in the course of disease.^2^ The Red Flags Index^3^ is an 8-item tool designed to help identify patients with Crohn’s disease, developed on a cohort of patients with either known Crohn’s or irritable bowel syndrome, as well as healthy volunteers. The recognized limitations of the Red Flag Index include not being subjected to external validation, and the use of a patient’s recollection of symptoms, which is subject to bias. In this issue of *CC360*, the architects of the IBD-REFER report on their effort to create an improved tool based upon both history and laboratory criteria for the identification of IBD in adults and children. Successfully employing both the Delphi method and multiple regression with sensitivity analysis, the IBD-REFER criteria demonstrated excellent operating characteristics in the internal, but also notably, in the external validation cohort, with sensitivity/specificity in adults reaching 98%/96% and 96%/96% in children. IBD-REFER outperformed the Red Flags Index when used on the same external validation cohort of IBD cases and controls, with respective ROC values of 0.97 vs 0.78 respectively (*P* < 0.001).

The IBD-REFER criteria are technically precise in the differentiation of known IBD patients from other gastrointestinal (GI) clinic patients without IBD, with relative ease of use. Each major criterion, for which only one element is required for referral, can be obtained from the patient’s stated history. The minor criteria, of which two are required for referral, are a mix of individual and family history, as well some laboratory criteria (elevated ESR/CRP, or ASCA/ANCA), which can also be easily obtained or are frequently already available. Notably, additional specialized testing like fecal calprotectin, imaging, or endoscopy are not part of the criteria, adding to the attractiveness of these criteria for ease of use. However, even in the external validation cohort of consecutive patients seen within a GI clinic, the same issues of recall and confirmation bias apply for patients with known diagnoses. Furthermore, the non-IBD controls in this cohort were carefully whittled down from 85 to 50 patients by excluding patients with specific other diagnoses, e.g. celiac disease and lactose intolerance, including 21 (25%) excluded for having additional other diagnoses. This was done with good intention to select an appropriate non-IBD comparison group, but potentially dilutes the true external validity should IBD-REFER be applied in its intended setting; for example, patients presenting to primary care with GI complaints. The performance of the IBD-REFER criteria in a more real-world setting, such as primary care or GI clinic patients without a known diagnosis, is unknown.

Finally, there are arguments and points of view to consider that apply to the use of any clinical tool. First is that many similar tools exist, including for other GI conditions, but few are used in routine clinical practice. This is probably due to a combination of usefulness in real-world situations, ease of use, and reliance on objective criteria. This likely explains the ubiquitous use of the Model for End Stage Liver Disease (MELD), consisting only of three common lab values, with use extending from its initial purpose of predicting survival after transjugular intrahepatic portosystemic shunt (TIPS), to now being central for liver transplant organ allocation, as well as predictive of alcoholic hepatitis survival and preoperative mortality.^4^ In contrast, despite the availability of multiple GI bleeding scores (AIMS65, Rockall, Glasgow Blachford), these are infrequently used in routine clinical practice. Arguably, this is because such scores predict mortality or re-bleeding, but are less helpful with decision making regarding the overall need for (e.g. to obtain a diagnosis) or relative timing of endoscopy. Such decisions are often the result of reliance on clinical judgement and experience within the local healthcare environment. Viewing the IBD-REFER criteria though this lens, the determination of who is likely to have IBD can be re-interpreted as clinical judgement; particularly the major criteria which are elements of patient history that would normally trigger a high degree of suspicion of IBD. In short, the IBD-REFER criteria seem to represent an attempt to quantify sound clinical judgment; perhaps making it most useful for those who have had less exposure to the diagnosis and care of patients with IBD.

In summary, the IBD-REFER criteria were rigorously developed with sound methodology, are easy to apply, and have excellent performance characteristics. However, significant limitations to its use include significant reliance on historic recall over objective findings. Additionally, validation was performed on persons with known IBD vs non-IBD diagnoses, and therefore subject to biases that potentially limit external, real-world performance. A prospective investigation of the IBD-REFER criteria in primary care or undiagnosed GI referral patients, with randomization to a comparison group that undergo clinical judgement/usual care, would be of great interest.
